# A protocol and a data-based prediction to investigate virus spillover at the wildlife interface in human-dominated and protected habitats in Thailand: The Spillover Interface project

**DOI:** 10.1371/journal.pone.0294397

**Published:** 2024-01-02

**Authors:** Chuanphot Thinphovong, Ewan Nordstrom-Schuler, Pipat Soisook, Anamika Kritiyakan, Ronnakrit Chakngean, Sakarin Prapruti, Malee Tanita, Yossapong Paladsing, Phurin Makaew, Awatsaya Pimsai, Abdulloh Samoh, Christophe Mahuzier, Serge Morand, Kittipong Chaisiri, Waraphon Phimpraphai

**Affiliations:** 1 Faculty of Veterinary Technology, Kasetsart University, Bangkok, Thailand; 2 Princess Maha Chakri Sirindhorn Natural History Museum, Prince of Songkla University, Songkla, Thailand; 3 Nanthaburi Nation Park, Thawangpha, Nan, Thailand; 4 Protected Areas Regional Office 13 (Phrae), Phrae, Thailand; 5 Primary Care Unit (PCU), Saenthong, Thawangpha, Nan, Thailand; 6 Institut d’Ecologie et des Sciences de l’Environnement de Paris (iEES Paris)—Centre de Recherche IRD, Montpellier, France; 7 MIVEGEC, CNRS–IRD–MUSE, Montpellier Université, Montpellier, France; 8 Faculty of Tropical Medicine, Department of Helminthology, Mahidol University, Bangkok, Thailand; 9 Faculty of Veterinary Medicine, Kasetsart University, Bangkok, Thailand; Sudan University of Science and Technology, College of Veterinary Medicine, SUDAN

## Abstract

The Spillover Interface Project aims at assessing the encounter of wildlife, domestic animals, and humans along a landscape gradient from a protected area to a residential community, through areas of reforestation and agricultural land. Here, we present the protocols of the project that combine virus screening in humans, bats, rodents and dogs with camera trapping, land-use characterization, and network analyses. The project is taking place in the sub-district of Saen Thong (Nan Province, Thailand) in collaboration with local communities, the District Public Health Office, and Nanthaburi National Park. To formulate a predictive hypothesis for the Spillover Interface Project, we assess the wildlife diversity and their viral diversity that could be observed in Saen Thong through a data science analysis approach. Potential mammalian species are estimated using data from the International Union for Conservation of Nature (IUCN) and their associated viral diversity from a published open database. A network analysis approach is used to represent and quantify the transmission of the potential viruses hosted by the mammals present in Saen Thong, according to the IUCN. A total of 57 viruses are expected to be found and shared between 43 host species, including the domestic dog and the human species. By following the protocols presented here, the Spillover Interface Project will collect the data and samples needed to test this data-driven prediction.

## Introduction

For several decades, the number of new emerging diseases as well as the number of epidemics of infectious diseases have continued to increase [1,2]. The vast majority of these diseases are zoonoses, involving domestic and/or wild animals [3]. The emergence of new zoonotic diseases begins with the spillover of a pathogen hosted in an animal reservoir [4], which is essential for maintaining the zoonotic agent [5]. The animal reservoir is assumed to be asymptomatic [6] and living in close relationship with humans [7]. Thus, emerging zoonotic diseases are primarily driven by interspecific interactions described as spillover events [8].

Spillover events are determined by a combination of biological and ecological processes in relation to hosts, pathogens, vectors and environmental conditions. Several studies have investigated the conditions that favor spillover [9,10] and, importantly, where a spillover has a greater chance to occur [6,11,12]. Spillover events may depend on various factors, from habitat change [13], climate variability [14], to social factors [15], which favor the encounter of reservoir and recipient hosts [16]. Habitat changes with agriculture intensification [17,18], livestock expansion [19], urbanization [20,21] and forest conversion [22] enhance the spillover and transmission of zoonotic diseases [23,24], often through generalist and synanthropic species [25]. The various combinations of these factors constitute the encounter interface.

An encountering interface can be classified as obvious or non-obvious. An obvious encountering interface is a location where interspecific transmission of a pathogen is predictable over time due to the continuous presence of both reservoir and recipient hosts. Wildlife markets or exploited caves for bat guano can be defined as obvious encountering interfaces. In contrast, non-obvious interfaces, such as ecotones related to human activities and rapid land use changes, can be characterized as a transient location with a narrow transmission and spillover opportunity due to short interspecific interactions [26,27]. This interface is difficult to identify because it requires ecological knowledge of the species of interest in order to assess their affinity with different habitats and their responses to environmental factors.

Among the new emerging viral zoonoses, bats harbor a high proportion of viruses [28–30], with 224 species of bats harboring 61 zoonotic diseases [29]. Rodents are also important, with 217 species harboring 66 zoonotic viruses [31]. Among these viruses, coronaviruses (CoVs) have received a great deal of attention [32]. In the last sixteen years, three coronaviruses of bat origin with high epidemic potential have emerged in human populations (SARS-CoV, MERS-CoV, and SARS-CoV-2) and one in domestic animals (SADS-CoV) [33]. Coronaviruses are also well known in veterinary medicine as they infect a wide range of mammalian hosts, such as Transmissible Gastroenteritis Coronavirus (TGEV) of domestic pigs [34], Mouse Hepatitis Virus (MHV) of mice [35], and Canine Coronavirus (CCoV) of dogs [36]. In Malaysia, Canine CoV (CCoV), a novel canine-feline recombinant alphacoronavirus was found in nasopharyngeal swab samples from eight out of 301 patients hospitalized with pneumonia, most of them children living in rural areas [37].

Reverse spillover, called spill-back, can occur from the human population to domestic animals or wildlife, as recently observed for SARS-CoV-2 in several mammal species [38], including white-tailed deer [39]. In Thailand, three dogs out of 35 examined were found infected by SARS-CoV-2 from households with confirmed COVID-19 residing patients [40]. The great diversity of coronaviruses is associated with their evolutionary capacity and ability to jump between species [41], which gives them an exceptional propensity for spillover and emergence [42].

The Spillover Interface Project aims at assessing virus sharing at the interfaces of wildlife, domestic animals, and humans along a gradient from a protected area to a village community and their agricultural land. The project will combine virus screening in various hosts in relation to land-use characterization [43,44].

Here, we present the objectives, design, and protocols used in the Spillover Interface Project. To formulate a predictive hypothesis for the Spillover Interface Project, we develop a predictive hypothesis based on open data to assess the wildlife diversity and associated viral diversity that can be observed in the implementing locality of the project.

## Materials and methods

### A. Spillover Interface Project design

#### Project objectives

The objectives are (1) to investigate the diversity of viruses, and in particular coronaviruses, in humans, domestic dogs, and wildlife (bats and rodents), (2) to identify the potential transmission of viruses among hosts using network analyses, (3) to identify potential interfaces using a land use map, (4) to assess the diversity of wildlife, their habitats and their potential role in virus spillover.

#### Location of the study

**Fig 1:**
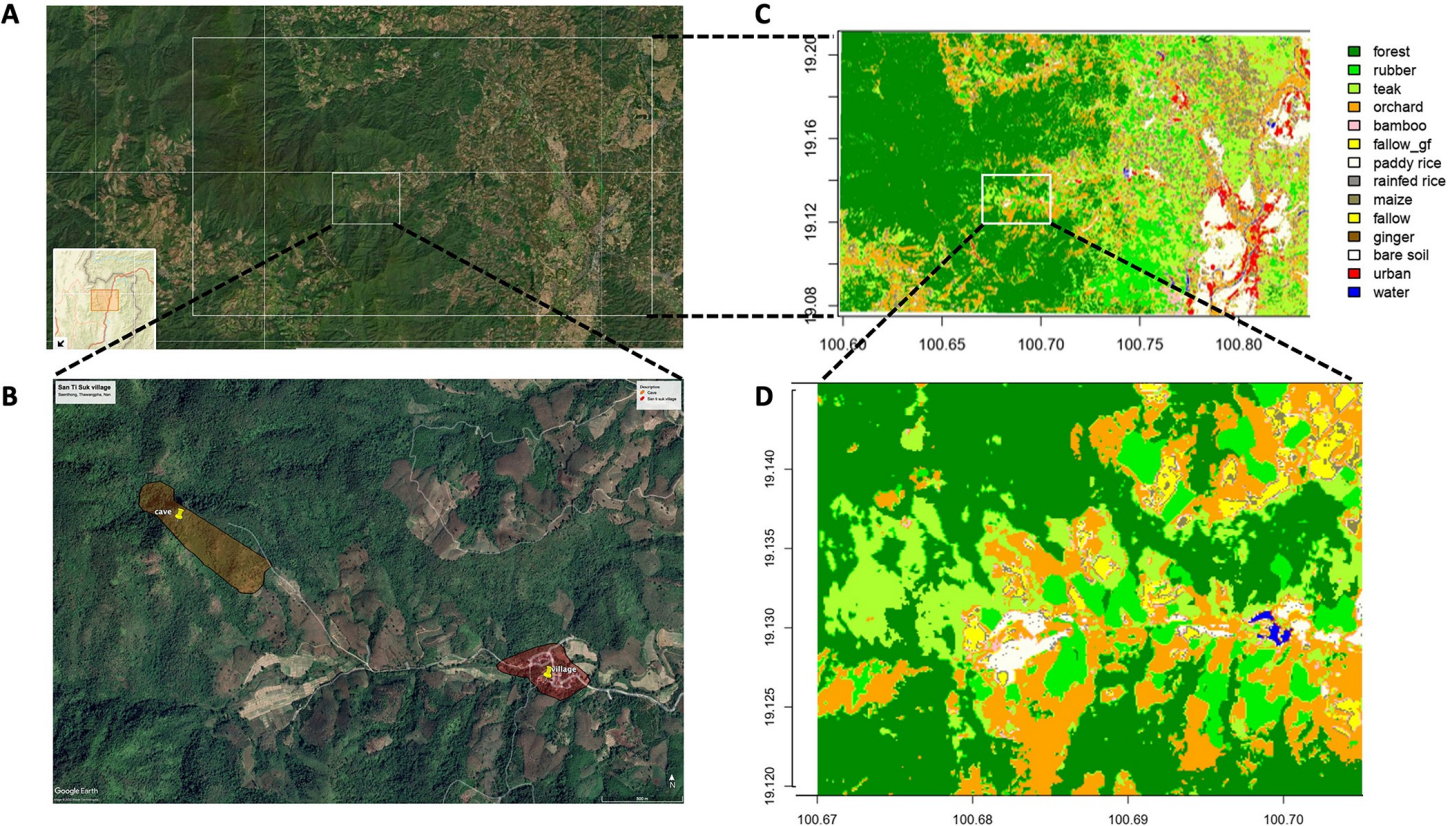
Location of the Spillover Interface project in (A) Saen Thong sub-district (Nan province, Thailand) precisely (B) in the upland part of the subdistrict. A land-use cover describes the different land classes: Multi-specific forests, plantations (rubber, teak, bamboo plantations, orchards), fallows crops (corn, paddy rice, ginger, etc.), urban infrastructural at (C) the level of the sub-district and (D) the upland part of the sub-district.

Since 2012, our research team has conducted several collaborative studies with local communities and local administrations, such as the District of Public Health Office and the Nanthaburi National Park in the sub-district of Saen Thong (Nan Province, Thailand). The sub-district is divided into two types of landscape, with a lowland agricultural zone (with four villages) close to an urbanized habitat and an upland agricultural zone (four villages) close to the forested area of Nanthaburi National Park [45]. The upland part of the sub-district provides an ideal site for the implementation of the Spillover Interface Project, with a gradient from the protected area of Nanthaburi National Park, with a cave populated by bats, towards the reforestation area, plantations, and agricultural land (Fig 1A and 1B).

#### Land use map

A high-resolution land use map (10 square meters) of Saen Thong subdistrict was developed for a previous project using Copernicus satellite data [46]. Random Forest classifications used the combination of Sentinel-2 optical and Sentinel-1 radar images with the different images acquired in 2019. The classifications cover four categories of lands: (1) uncultivated steep mountain slopes with forest (protected areas and community forests), (2) cultivated land mostly on steep slopes with annual crops (such as upland rice and corn), or plantations (such as rubber, teak and bamboo), (3) cultivated lowland with paddy fields, (4) urbanization and infrastructures. All farmers are smallholders, and field sizes are relatively small. The classifications were validated by ground-truth checks [46].

The land use classification makes it possible to differentiate multi-specific and heterogeneous forests (mixed forests), including reforestation areas and community forests, from plantations such as rubber, teak, bamboo or orchards. The land use also distinguishes dominant crops (corn, paddy rice, ginger, etc.), houses, and other infrastructure (Fig 1C and 1D). A terrain elevation model was also developed [46]. This land use map is important (1) to assess the typology of the different habitats of the locality allowing statistical comparisons and (2) to describe the behavior of animals at high resolution, ie. the movement or home rage of animals in a small buffer zone around each camera trap or live trap by tags for rodents or pictures for dogs and other wildlife when individual recognition is possible.

#### Framework

**Fig 2:**
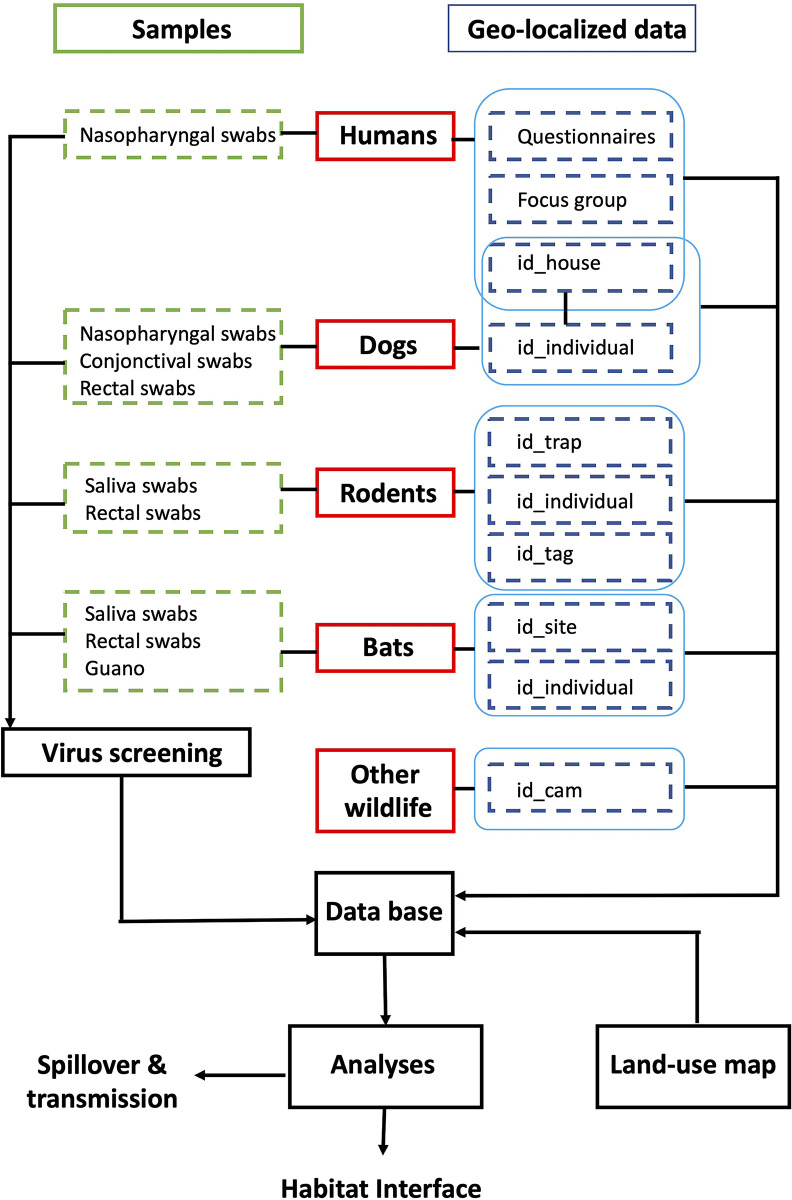
Framework for sampling, swabs collected for virus screening, and data collected from questionnaires and focus groups from villagers, from rodent and bat trappings and from camera trappings (with associated GPS locations for each device). Data will be collected in the field using a data form designed from the Epicollect5 application installed on smart phones.

A framework was designed for sampling and data collection in order to validate the predictive hypothesis. Rodents will be captured by live traps and bats will be trapped by mist nets and harp traps. Sample collection will include all swabs and guano samples collected for virus screening. Data collection will include qualitative data obtained from questionnaires and focus group discussions with villagers, as well as quantitative data such as weight and body measurements obtained from rodents and bats during the trapping sessions. GPS coordinates will be recorded at individual traps and camera trapping devices. In the field, all information will be collected digitally (Fig 2).

#### Research and ethical approvals

The principles of ethics and responsibility require local community and stakeholder engagement, such as in the Primary Health Care Unit, the District Public Health Office, and Nanthaburi National Park. Several meetings with all stakeholders helped to co-construct the project which made it possible to meet the objectives of human health, animal health, and biodiversity conservation.

Procedures for human health investigation, laboratory investigation, interviews, and questionnaires will be sent for approval by the ethical committee of the Nan Provincial Public Health Office, Ministry of Public Health, Thailand (NAN REC 63–13). All participants who agree to join the study will be asked to read the Participant Information Form, which explaining the objectives, procedures, possible risks, and benefits of the research project.

Similarly, procedures for collection of samples from dogs, laboratory investigation, safety procedures, interviews and questionnaires of dog owners will be sent for approval by the Institutional Animal Care and Use Committee, Kasetsart University (ACKU64-VTN-010).

Rodent and bat species that will be investigated by Spillover Interface Project will be neither on the CITES list nor on the Red List (IUCN). Animals will be treated in accordance with the guidelines of the American Society of Mammalogists, and within the European Union legislation guidelines (Directive 86/609/EEC). Any trapped rodent species listed on CITES will be released without being manipulated. All animals will be released after sampling. Approval notices for trapping and investigation of bats and rodents will be sent for approval by the Institutional Animal Care and Use Committee, Kasetsart University.

Permit has been approved by the Department of National Parks, Wildlife and Plant Conservation (DNP) and the Royal Forest Department, Ministry of Natural Resources and Environment, Thailand. In addition, foreign researchers participating in the research project have also been approved by the National Research Council of Thailand (NRCT) with the consent of the DNP.

With community engagement, ethics, and research permits, the investigation will begin with the organization of a meeting involving the researchers, local public health workers, the village leaders, and village volunteers to remind them of the purpose and details of the study. Interviews with villagers will help to describe their interactions with wildlife. Moreover, it will help to select participants who will express close contact with wildlife, either bats or rodents.

### Field implementation and virus screening protocols

#### Bat sampling

Bats will be sampled once inside a cave situated in Nanthaburi National Park and in the village of Santisuk using mist nets and harp traps [47]. Traps will be set before sunset and checked after sunset depending on bat activity time. Measurements will be taken for each captured bat: weight, forearm, hindfoot, tail, ear, head to body, and tibia lengths. Saliva and rectal swabs will be collected, and stored in RNA later and kept at -20°C. For other sessions, only guano will be collected from the cave.

#### Rodent sampling

The rodent trapping is designed to cover a gradient from the village to the bat cave situated in the Nanthaburi National Park area. Rodent trapping will then cover several habitat types, including agricultural crops, plantations, and the reforestation area at the interface between plantations and the National Park. Live-traps, locally made (dimension: 14 X 14 X 27 cm) will be used. A total of 210 traps will be set: 40 traps in and around the village, 60 traps in agricultural land and plantations, 100 traps in the reforestation area, and 10 traps inside the cave. Traps will be set in lines of five traps separated by a distance of 10 meters. The different lines will be set to evenly cover the gradient. Trapping will be conducted over a period of four nights for each session, which corresponds to a total of 840 trap nights per trapping session. The traps will be placed in the same positions for each session by using plastic plant labels as notification tags on the ground. Pictures, habitat descriptions and rodent trap coordinates available for the CERoPath Project (https://healthdeep.shinyapps.io/Small_mammals_CERoPath/) will be used for rodent species identification [44].

Rodents will be anesthetized using a mixture of 20% v/v isoflurane in propylene glycol. Two ml of mixed isoflurane will be placed on cotton or gauze in a transparent plastic box chamber (32 X 22 X 15 cm). The individual animal will be introduced into the chamber, and respiratory rate will be observed after anesthetizing for one minute in the case of a mouse-size rodent and for two minutes for a rat-size rodent [48,49]. The animal will be removed from the anesthetic chamber, its weight will be recorded, and pictures will be taken for further body measurements. Saliva and rectal swabs will be taken, and stored in RNA later and kept at -20°C. A microchip (Passive Integrated Transponder: PIT Tag) of 1.4 X 8 mm will be injected subcutaneously for individual permanent identification and for capture-mark-recapture (Absonutrix Sale Company, ISO11784/785). Captured animals will be read with a microchip reader (Absonutrix Sale Company, mini scanner ISO FDX-B 134.2 KHz) to evaluate re-trapping or new trapping. Rodents will be carefully monitored before releasing them at their trap location captures. Any re-trapped individual during a session will be released without manipulation.

#### Dog sampling

Most dogs roam freely in the upland villages of Saen Thong and may go to agricultural or reforestation areas with their owners. Free-roaming dogs will be selected with the help of the village leader, who will invite dog owners to freely join the research project. The participant dog owners will take their dogs to the village hall, where we will collect nasopharyngeal swabs, conjunctival, swabs and rectal swab with the consent of their owners. Nasopharyngeal, conjunctival and rectal swabs that will be placed in cryotube contained 0.5 μl of RNA later, will be stored in RNA and kept at -20°C. Conjunctival swabs will be used to screen asymptomatic [50] as well as symptomatic cases of COVID-19 [51].

#### Virus screening

For RNA extraction, the QIAamp Viral RNA Mini Kit (Qiagen) will be used following the manufacturer’s instructions. The extracted RNA will be measured for concentration, purity and quality checking using Nanodrop [41]. Concerning CoVs, extracted RNA will be used for reverse transcriptase (RT) reactions using SuperScript III Reverse Transcriptase (Life tech) and hexamers with primers and semi-nested PCR protocols following Gouilh et al. [43]. We will keep extracted DNA and RNA extraction for further viral analyses [52].

#### Camera trapping

Camera trapping helps to investigate wildlife diversity along the gradient from the protected area, especially species visiting the bat cave, to the reforestation area and agricultural land. A total of 40 camera traps will be positioned following a land use map illustrating forest or reforested areas, rodent trapping lines, and suggestions from the rangers of Nanthaburi National Park and the village volunteers based on their ecological knowledge. The camera traps will be left in place for a year. The camera traps will be checked every two months. Batteries will be changed, and pictures taken by the camera traps will be transferred to a laptop computer. Later, the pictures will be sorted, and the identification of species will be assessed by a consensus of experts. The sorted pictures by species and by camera traps will be analyzed using the ‘camtrapR’ package [53] implemented in R, which will allow the exploration of the spatio-temporal activities of animals, including roaming dogs.

### B. Predictive hypothesis

#### Potential wildlife species and viruses

We developed a predictive modeling that helps formulate hypotheses to be tested by the Spillover Interface Project, such as which mammal species and which viruses can be found in the study locality. To describe the potential association between mammal species and virus, we estimated the potential presence of mammal species in the study site using data from the International Union for Conservation of Nature (IUCN). Shapefiles of terrestrial mammal species were downloaded from the IUCN Red List (https://www.iucnredlist.org/resources/spatial-data-download) providing geographical distribution for each animal species. The IUCN Red List the status of each mammal species and their CITES status. The list of mammal species from the orders Carnivora, Cetartiodactyla, Chiroptera, Pholidata, Rodentia, and Scandentia, that are potentially present in Saen Thong was then extracted using the land use map presented above. Animal species were identified on the basis of their overlap distribution, given by the IUCN shapefiles, with Saen Thong area given by the land-use map [46]. Concerning the viral diversity, we used an open database of nucleotide sequences published between 1950 and 2019 compiling the associations between 1,785 virus species (DNA and RNA) with 725 mammalian host species that resulted from the automatic screening of the accompanying metadata [54,55].

#### Network analyses

We used a network analysis approach to represent and quantify the ecology of virus transmission between different hosts [56], as it allowed representation and mathematical quantification of the importance of a given host in the transmission of viruses. Viral diversity and potential transmission to humans were assessed using network architectures and associated indices. In bipartite networks, only host–virus interactions are considered, representing how host species and virus species are associated. Viruses and hosts represent nodes of the network, and edges represent the observed interactions between nodes in the network. In a unipartite network, nodes represent the hosts and edges the viruses shared between each node (i.e. host).

Modularity in bipartite and unipartite networks of shared viruses help assess host communities sharing similar virus transmission and then potential risks of transmission of other viruses between species [57]. Modularity measures the strength of division of a network into modules, i.e. groups or clusters of hosts sharing common viruses.

Network centrality indices provided useful information on the relative importance of a given host to the entire network [58]. A host occupying a highly central position (i.e., having a high centrality value) links different hosts clustered into subgroups within the network and participates strongly in the transmission among a large community of hosts. Hosts with high values of centrality in networks can help target key reservoirs for viral disease surveillance [54,56,57].

We first linked the list of mammal species extracted from the IUCN, adding the domestic dog (*Canis lupus familiaris*) and the human species (*Homo sapiens*), to the dataset of virus species from mammals [47]. Using the information linking each host species with their virus, we obtained bipartite networks and unipartite projections in which each node was a host species. Modules were identified for bipartite and unipartite networks of shared viruses between mammal species. We used bipartite network analysis, with nodes describing the mammal species interacting with nodes describing the viruses using the ‘bipartite’ package [58,59] implemented in R [60]. We used the function ‘computeModules’ of the package ‘bipartite’ to compute modules using the modularity algorithm of Dormann & Strau [61]. We projected these bipartite networks onto unipartite networks using the ‘tnet’ package [62] implemented in R. A unipartite network represented relative interactions amongst hosts through the sharing of virus species. Each host within the network played a different role in virus sharing relative to all other nodes, which was examined using its centrality measurement. We used the function ‘cluster_louvain’ implemented in the package ‘igraph’ [63] to identify the modularity structure of the unipartite networks. This function is based on a multilevel modularity optimization algorithm [64]. A central node (i.e. a host with a high centrality value) was the one that was highly connected to other nodes and thus was supposed to have a greater transmission potential for virus species. We calculated the eigenvalue centrality (EC) with the ‘evcent’ function from the package ‘igraph’ [63].

## Results

**Fig 3:**
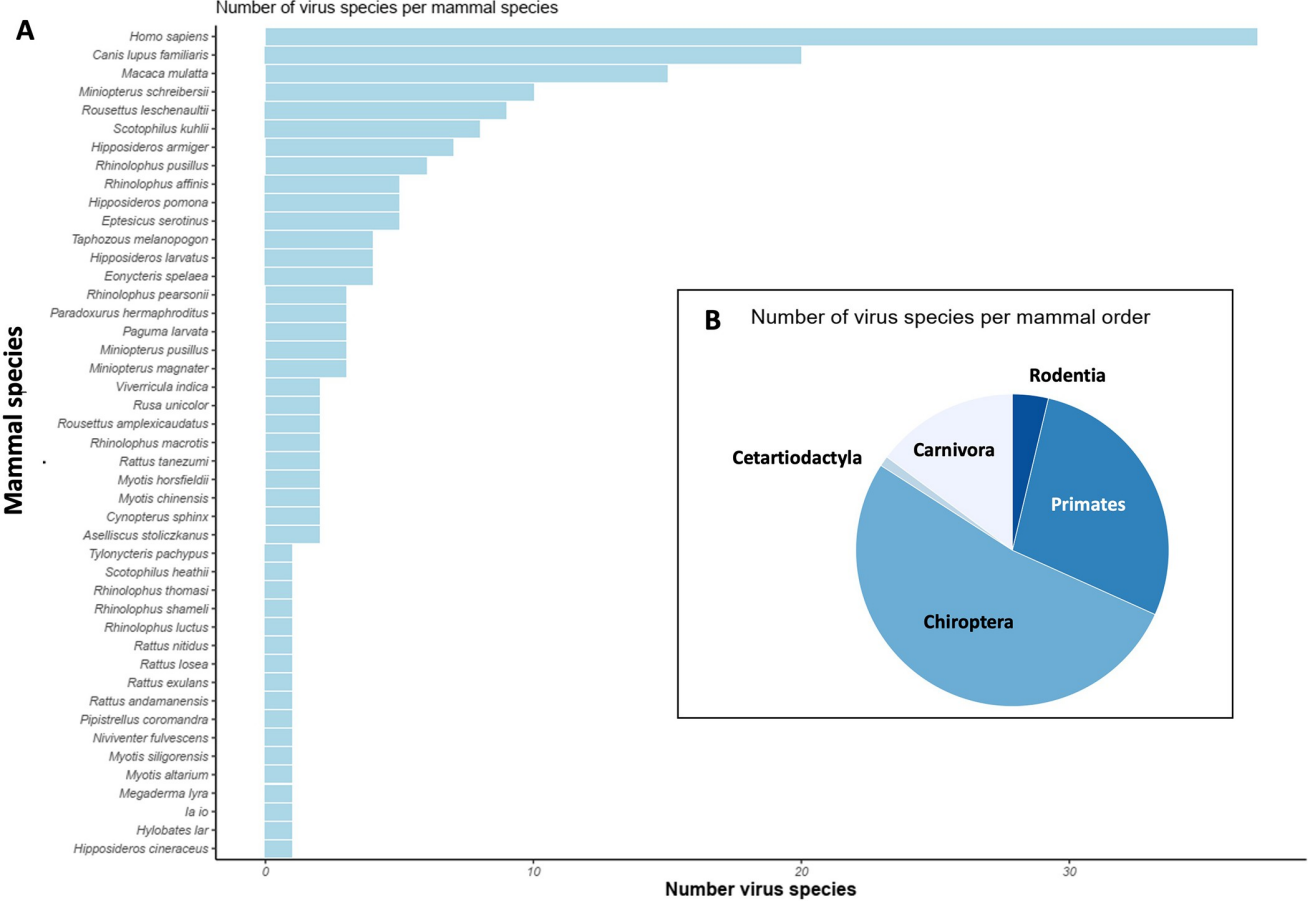
Diversity of potential viruses hosted in species potentially present in Saen Thong with (A) the number of viruses per mammal species including the human species and the domestic dog and (B) the number of viruses per mammal orders. Data on the potential presence of wild mammals were extracted from the IUCN Red List and data on the potential viruses from an open database.

The predictive modeling used network analysis to explore the association between mammal species and their viruses. A total of 130 mammal species are potentially present in Saen Thong sub-district according to the IUCN Red List, although we removed several species known for their absence in the area (such as *Mus musculus* and *Rattus rattus*). The species were checked and confirmed using the CERoPath database and the data collected by the Smart Patrol Project of Nanthaburi National Park. This list of mammal species, also including the domestic dog (*Canis lupus familiaris*) and the human species (*Homo sapiens*), was linked to the dataset of virus species from mammals. A total of 57 viruses were found to be shared between 43 host species (see S1 and S2 Tables), including the domestic dog and the human species (Fig 3A), with the human species hosting the highest number of viruses followed by the domestic dog, the Indochinese rhesus macaque (*Macaca mulatta*), and several bat species. Bats showed the highest diversity of viruses shared among the whole host communities, followed by human and non-human primates, carnivores, rodents, and cetartiodactylids (Cervidae) (Fig 3B).

**Fig 4:**
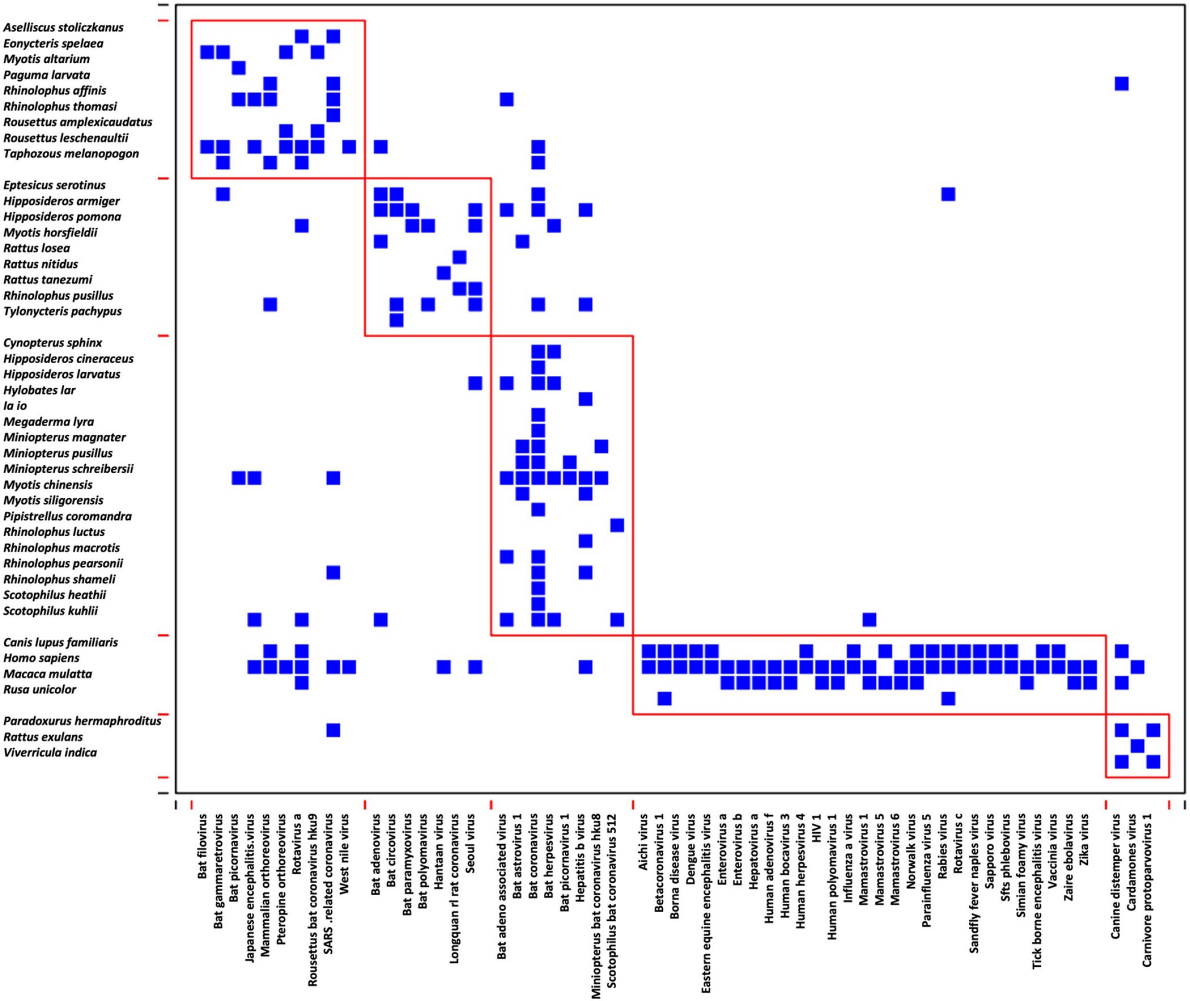
Bipartite network of potentially shared virus species among wild mammal species potentially present in Saen Thong and the human species and the domestic dog. Five modules were identified with one the one grouping humans, domestic dog, Indochinese rhesus macaque, and the Sambar deer with ten viruses. Bat species were separated in three modules, and rodent species in two modules.

**Fig 5:**
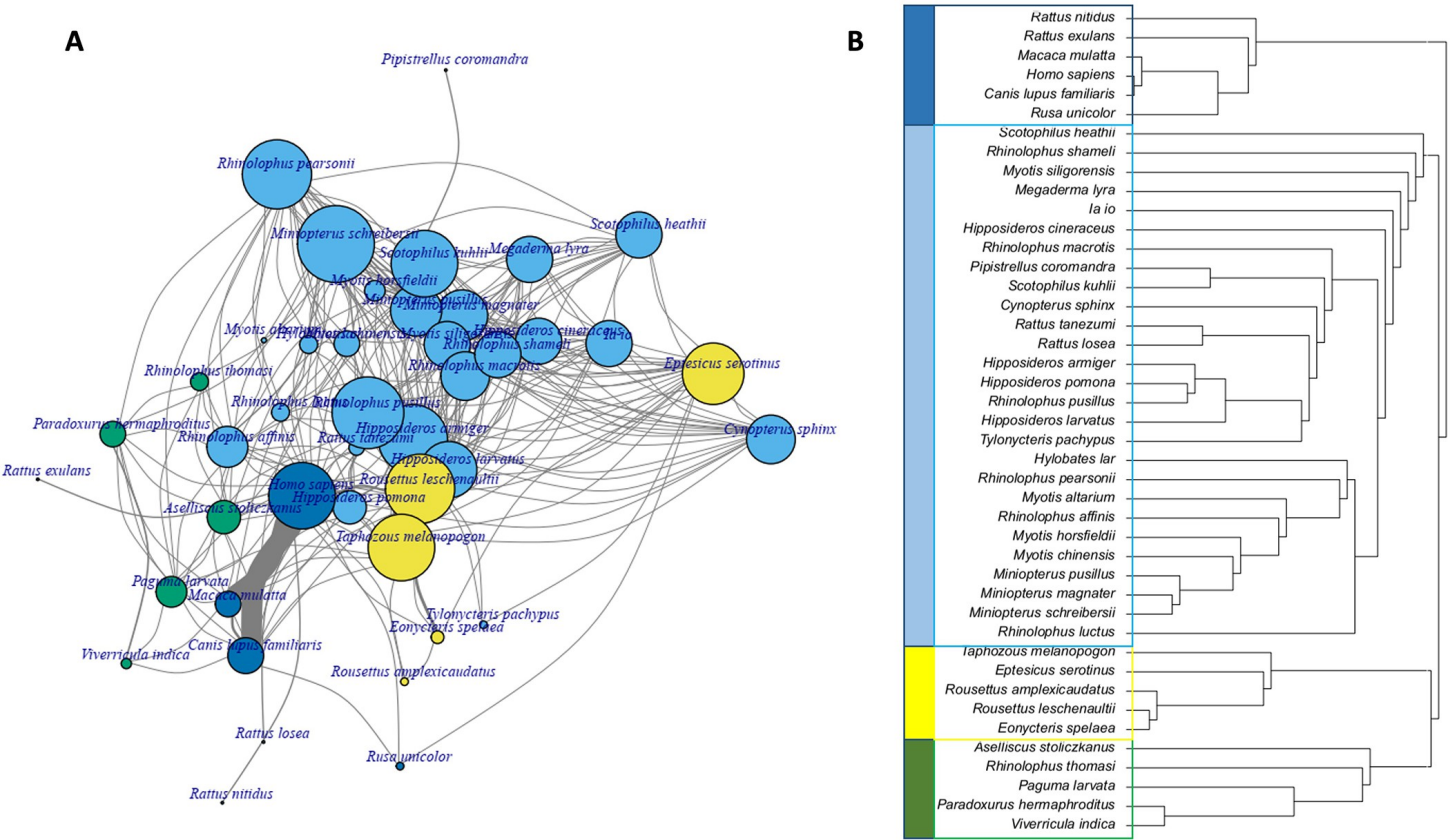
(A) Unipartite network with modules differentiated by colors of shared virus species among mammal species including humans and domestic dog. The links among hosts (nodes) of the unipartite network depict shared viruses (vertices were placed according to the Fruchterman–Reingold algorithm) with thickness of links proportional to number of viruses shared and size of vertices proportional to the degree centrality of hosts. (B) Clustering representation of the unipartite network with the different clusters differentiated by colors. Four modules were identified with one grouping the human species with the domestic dog, the Indochinese rhesus macaque, the Sambar deer and two species of rodents (*Rattus exulans*, *Rattus nitidus*). The bat species were separated in three modules, with one comprising two rodent species (*Rattus tanezumi*, *Rattus losea*), and one comprising three carnivore species (Paguma larvata, Paradoxurus hermaphroditus, Viverricula indica).

Bipartite networks and unipartite projections were obtained using the information linking each host species with their virus. Modules identified for bipartite and unipartite networks differed by their numbers of modules (Figs 4 and 5). Using the bipartite network, five modules were identified (Fig 4), such as the one grouping the human species, domestic dog, Indochinese rhesus macaque, and Sambar deer (*Rusa unicolor*) with ten virus species. The bat species were separated into three modules, and the rodent species into two modules.

Four modules were identified using the unipartite network. The first module grouped the human species with domestic dog, Indochinese rhesus macaque, Sambar deer, and two species of rodents (*Rattus exulans*, *Rattus nitidus*). The bat species were separated into three modules, with one comprising two rodent species (*Rattus tanezumi*, *Rattus losea*), and one comprising three carnivore species (*Paguma larvata*, *Paradoxurus hermaphroditus*, *Viverricula indica*) (Fig 5).

**Fig 6:**
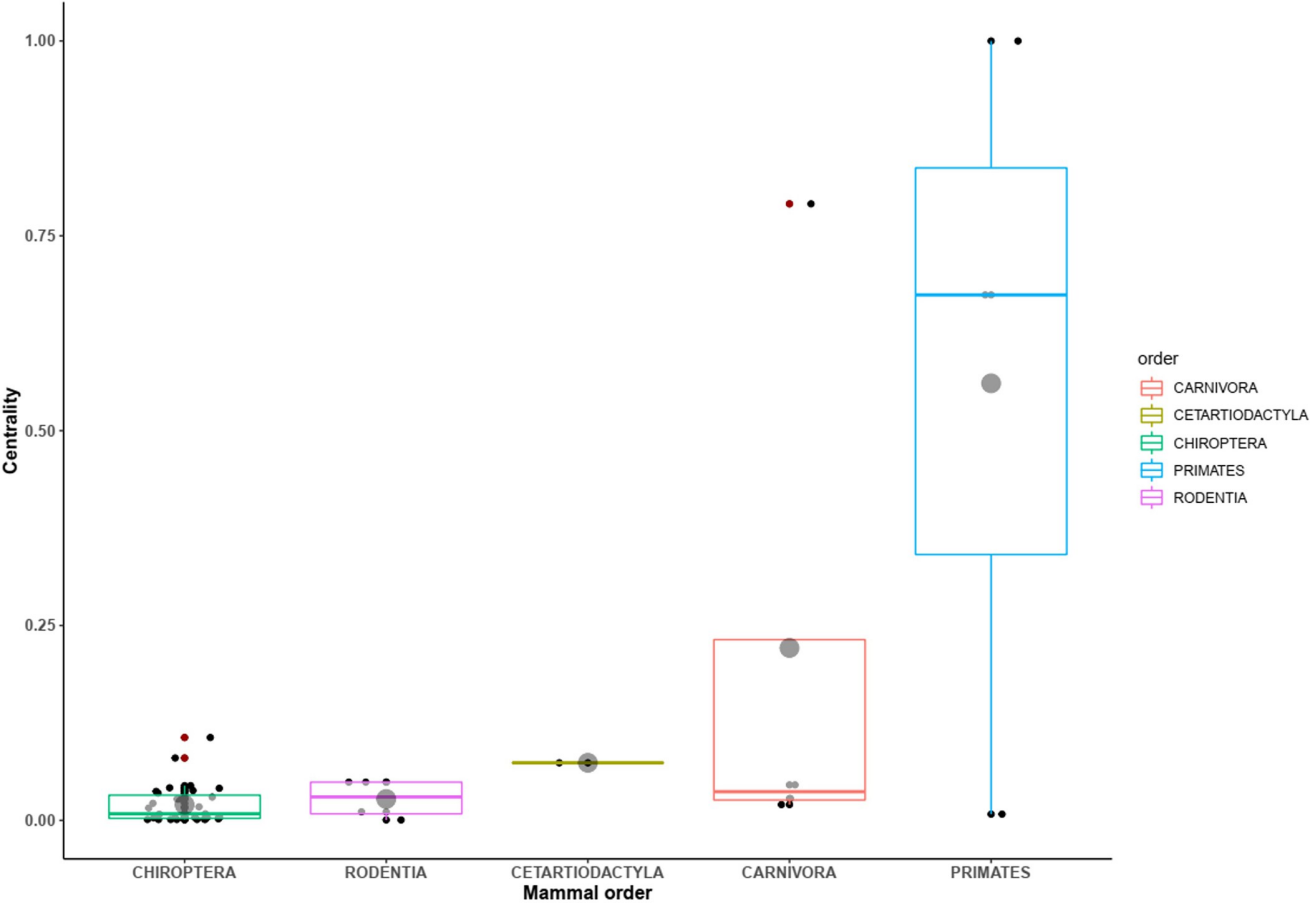
Box and whiskers plots of host centrality measures for sharing virus. Notched boxes for orders of mammals correspond to 95% confidence interval (horizontal lines inside the boxes are the medians of the centrality values, grey dots are means of centrality values, standard deviation are represented by vertical lines).

In terms of host centrality in the network of virus sharing, the primates showed the highest centrality because of the human species, followed by carnivores due to the domestic dog, the deer species (Cetartiodactylida), the rodents, and the bats (Fig 6).

## Discussion

Pathogen spillover events depend on various factors that favor the encounter of reservoir hosts and recipient hosts. Encountering interfaces is not always easy to characterize, mostly because of the lack of ecological knowledge about the species of interest. The protocols of the Spillover Interface Project will combine pathogen screening in humans, domestic dogs, bats, and rodents with extensive camera trapping, land use characterization, and network analyses in order to assess the encountering of wildlife, domestic animals and humans.

Using a data-based approach, we have made predictions about the wildlife diversity and viral diversity that can be observed in Saen Thong. The use of IUCN and virus open databases helped to predict the potential diversity of virus–mammal interactions in the locality. Using network analysis, we showed the importance of bat species as major reservoirs of potential zoonotic viruses. However, carnivores, along with domestic dogs, appeared central in the virus sharing among all mammal species [57,65]. The predictive analysis using open access data and network analysis showed the importance of several species. Synanthropic rodents and the domestic dog appeared central in the network of sharing viruses among humans and wildlife species that are potentially present at the location site. This data-based prediction confirmed the importance of investigating not only bats and rodents but also the domestic dog, which are then the targeted species of the Spillover Interface Project.

The results of this data-based prediction will be interrogated with the data gathered by the Spillover Interface Project. First, with the data on the diversity and co-occurrence of wildlife species in the different habitats gathered by the camera and live traps. The land-use map will allow the precise identification of habitat use and shared by each species trapped or pictured, and potential interfaces. Second, the screening of viruses will permit the investigation of their diversity and the number of species that individual viral species are able to infect such as small terrestrial mammals, bats, dogs, and importantly, across habitats. Last, the trapping design will standardize the sampling effort to assess the presence and abundance of the investigated small mammals and domestic dogs in each habitat.

## Conclusion

We presented the results of a predictive modeling approach illustrating the potential presence and association of hosts and their viruses in Saen Thong using network analysis. The protocols and framework of the Spillover Interface Project were designed to test the results of the predictive modeling in the Spillover Interface Project, first starting with coronaviruses.

## Supporting information

S1 Table The list of mammal species in Saen Thong, Thawangpha, Nan Thailand that was reported in the International Union for Conservation of Nature (IUCN). (CSV)

S2 Table The list of viruses which link to mammal species in Saen Thong, Thawangpha, Nan Thailand. Their associated viruses from a published open database.
(CSV)
